# Developing a prediction model to estimate the true burden of respiratory syncytial virus (RSV) in hospitalised children in Western Australia

**DOI:** 10.1038/s41598-021-04080-3

**Published:** 2022-01-10

**Authors:** Amanuel Tesfay Gebremedhin, Alexandra B. Hogan, Christopher C. Blyth, Kathryn Glass, Hannah C. Moore

**Affiliations:** 1 Wesfarmers Centre of Vaccines and Infectious Diseases, Telethon Kids Institute, University of Western Australia, Perth, 6872 Australia; 2 MRC Centre for Global Infectious Disease Analysis, School of Public Health, Imperial College London, London, UK; 3 School of Medicine, The University of Western Australia, Perth, WA Australia; 4 Department of Infectious Diseases, Perth Children’s Hospital, Perth, WA Australia; 5 PathWest Laboratory Medicine, QEII Medical Centre, Nedlands, Perth, WA Australia; 6 Research School of Population Health, Australian National University, Canberra, Australia

**Keywords:** Epidemiology, Viral infection

## Abstract

Respiratory syncytial virus (RSV) is a leading cause of childhood morbidity, however there is no systematic testing in children hospitalised with respiratory symptoms. Therefore, current RSV incidence likely underestimates the true burden. We used probabilistically linked perinatal, hospital, and laboratory records of 321,825 children born in Western Australia (WA), 2000–2012. We generated a predictive model for RSV positivity in hospitalised children aged < 5 years. We applied the model to all hospitalisations in our population-based cohort to determine the true RSV incidence, and under-ascertainment fraction. The model’s predictive performance was determined using cross-validated area under the receiver operating characteristic (AUROC) curve**.** From 321,825 hospitalisations, 37,784 were tested for RSV (22.8% positive). Predictors of RSV positivity included younger admission age, male sex, non-Aboriginal ethnicity, a diagnosis of bronchiolitis and longer hospital stay. Our model showed good predictive accuracy (AUROC: 0.87). The respective sensitivity, specificity, positive predictive value and negative predictive values were 58.4%, 92.2%, 68.6% and 88.3%. The predicted incidence rates of hospitalised RSV for children aged < 3 months was 43.7/1000 child-years (95% CI 42.1–45.4) compared with 31.7/1000 child-years (95% CI 30.3–33.1) from laboratory-confirmed RSV admissions. Findings from our study suggest that the true burden of RSV may be 30–57% higher than current estimates.

## Introduction

Respiratory Syncytial Virus (RSV) is a leading cause of morbidity and mortality in young children worldwide, causing 3.2 million detected hospitalisation episodes every year^1^. The true burden is likely to be much greater, with approximately half of RSV-associated deaths estimated to occur outside of hospital^1^. In Australia, for every 100,000 hospitalised children aged < 5 years, an estimated 418 have RSV^2^. A recent population-based study in Western Australia (WA) conducted by our group reported pathogen-specific incidence rates of 247/100,000 child-years for RSV in children aged < 17 years^3^, with the highest burden among infants in their first 3 months of life (28.1/1000 child-years)^4^. RSV is most frequently associated with hospitalisations for acute bronchiolitis, but was also identified across other clinical diagnoses including pneumonia, unspecified acute lower respiratory infections, asthma, upper respiratory infections as well as non-specific viral diagnosis codes^3,5^.

Population-wide in our jurisdiction and elsewhere, there is no systematic approach to RSV testing, nor is it currently a notifiable disease, making estimates of RSV disease burden using microbiological testing datasets alone difficult. From our population-based study, only 10% of children aged < 17 years were ever tested for RSV and variable testing trends were seen between age groups and from year to year. Additionally, 54% of children hospitalised with respiratory infections did not have a corresponding microbiological test^3^. Improved estimates of RSV burden are required to inform future policy for RSV therapeutics and preventative strategies, as late-stage clinical trials of antivirals, maternal vaccines and monoclonal antibodies progress^3,6^.

Understanding the demographic and clinical predictors of RSV test positivity in different populations can aid in quantifying the under-ascertainment burden of RSV from standalone datasets. Ideally, a combination of clinical and laboratory data is needed. Such a study has been conducted in England for infants aged < 1 year, using hospitalisation data and RSV positive testing records^7^.

The aim of our study was to develop a prediction model to estimate the true incidence of RSV associated hospitalisations in children < 5 years of age in WA and to use these findings to determine the under-ascertainment fraction of RSV incidence using laboratory records alone.

## Methods

### Setting and data sources

WA covers the western third of Australia with a population of approximately 2.6 million people at 31 December 2019^8^. Three quarters of the population reside in the temperate climatic region of metropolitan Perth and its surrounds^9^. We conducted a population-based cohort study using administrative linked data of all births in WA (1996 to 2012), as previously reported^3,10^. Data sources used for this study included the Midwives Notifications System, which includes perinatal information on > 99% of births in WA^11^, Birth and Death Registries, Hospital Morbidity Data Collection and PathWest Laboratory Medicine Database (PathWest). Data were probabilistically linked using best practice protocols through the WA Data Linkage Branch^12^.

#### Hospital data

Hospitalisation records, herein referred to as hospital admissions, with an admission and discharge date between 1 January 2000 and 31 December 2012 were included, to match the same time period when routine laboratory data were available. We included all admissions in children aged < 5 years in WA with any diagnosis using *International Statistical Classification of Diseases and Related Health Problems,10th Revision, Australian Modification* (ICD-10-AM) codes. As per previous analyses of these data, interhospital transfers were collapsed^3,10^.

#### Laboratory data

**Figure 1:**
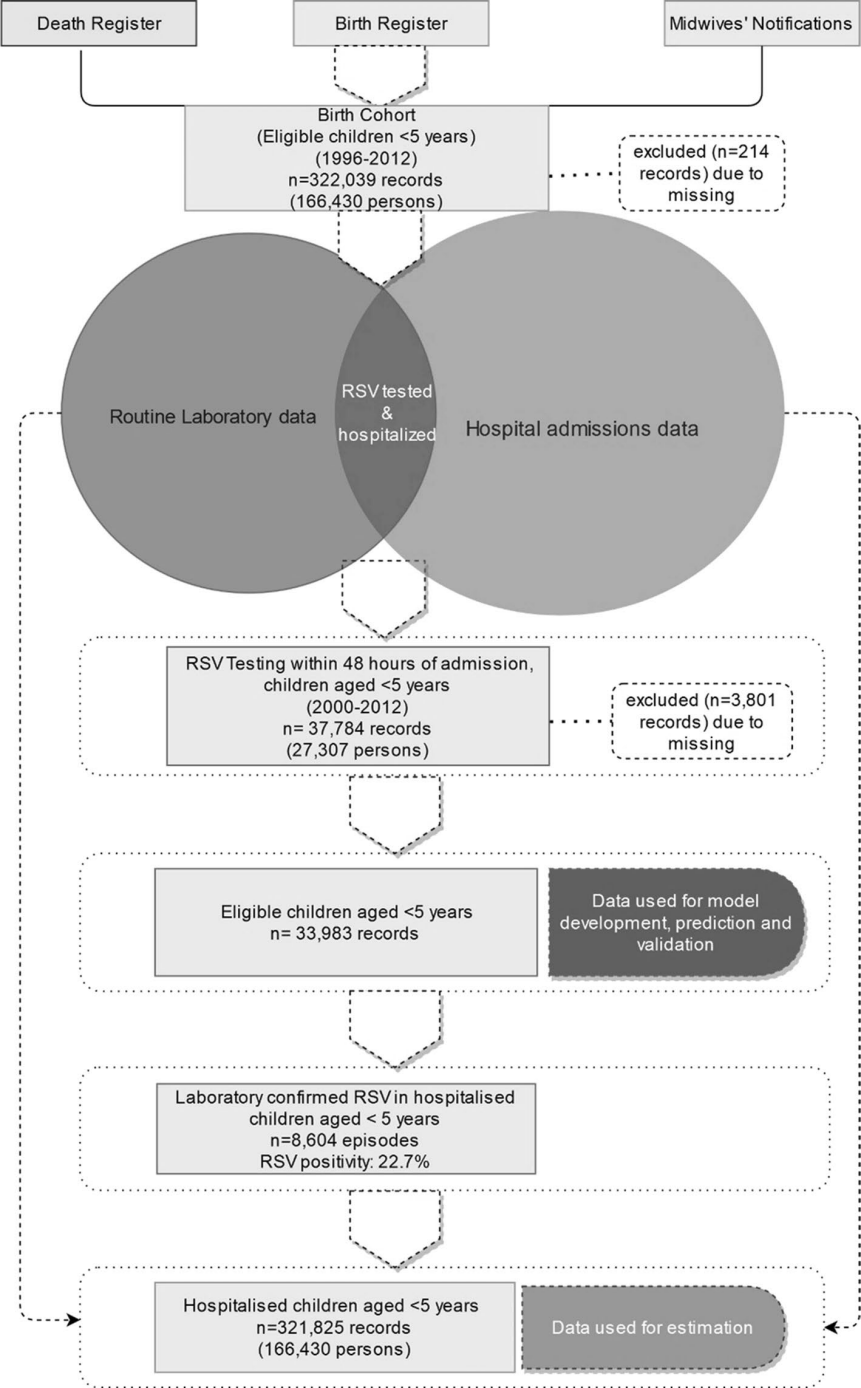
Flow diagram of datasets.

We extracted PathWest testing records of RSV from respiratory specimens with a specimen collection date between 1 January 2000 and 31 December 2012. We then linked these records with hospitalisation records from individuals from the birth cohort when respiratory specimens were collected 48 h before or after the admission date, as per our previous analyses^3,10^. The laboratory records were linked to the admission closest to the date of specimen collection when the same child had multiple admissions for different reasons within 48 h. During the early study period, RSV was predominantly detected through immunofluorescence antigen detection (65%) and viral culture on respiratory specimens and complement-fixation tests (CFT) on serum while, gradually, polymerase chain reaction (PCR) on respiratory specimens was more frequently used^4,10^. Using assembled data from hospital and PathWest records, we then identified our source population defined as children in the cohort who had a hospitalisation for any reason in the first 5 years of life with RSV testing records during the study period (n = 37,784 hospitalisations). After exclusion of 3,801 records with missing data for one or more of the variables, we finally included 33,983 hospitalisations with RSV testing records in the final prediction model. Similarly, after excluding observations with missing data, we estimated RSV burden in 321,825 records of hospitalised children under 5 years of age during the study period (Fig. 1).

### Statistical analysis

#### Developing prediction models

A multivariable logistic regression model was fitted to identify predictors of RSV-positivity (binary outcome) amongst children younger than 5 years who were hospitalised and tested for RSV in WA during the study period (2000–2012). We used a robust standard error estimation adjusting for correlated observations due to children having multiple admissions over the study period^13^. A total of 27 candidate predictors for the prediction models were selected based on a comprehensive literature review, including previous work in our setting^14^ and clinical plausibility.

Directed acyclic graphs (DAG) were used to inform the choice of these predictors. In modern epidemiology, DAGs are used as a tool in causal inference to identify and demonstrate knowledge, theories and assumptions about causal relationships between variables^15,16^. Causal inference principles have recently been more widely applied in prediction models^17,18^. The minimal set of variables identified by DAGs included child age, sex, delivery route (vaginal vs caesarean), season of admission, Aboriginal and/or Torres Strait Islander status, respectfully referred to as (Aboriginal/non-Aboriginal), as identified through a validated algorithm provided by the WA linkage branch^19^, maternal age (categorised), smoking during pregnancy (yes/no), socioeconomic status (in quantiles), numbers of siblings, gestational age (< 32 weeks, 32–36 weeks and 37 or more weeks), admission year, birth year, length of hospital stay (continuous), admission to intensive care unit (ICU) [binary], mechanical ventilation use during hospital admission (see below), maternal history of asthma (binary), any diagnosis code for acute bronchiolitis, pneumonia, bronchitis, unspecified acute lower respiratory infections (ALRI), asthma, URTI, whooping cough, influenza and diagnosis code for other infections including unspecified viral illness, convulsions, fever and cough, breathing abnormalities (refer to eTable 1). These specific codes were chosen based on our previous work that identified a positive detection of a respiratory virus in hospital admissions with these codes^10^. Further, we included season of birth and geographical region of residence to account for seasonality and climatic conditions (as a proxy) respectively (eTable 1). Our final prediction model also included a sine and cosine function as per the recommendation by Stolwijk and Edwards to account for the seasonal variation of RSV^20,21^.

Socioeconomic status (SES) was measured using Socio-Economic Indexes for Areas (SEIFA). Specifically, we used the Index of Relative Advantage and Disadvantage score, calculated at the collection district (CD) level by Australian Bureau of Statistics^22^.

A child was coded as admitted to an ICU if they spent at least one day in ICU as recorded on the Hospital Morbidity Data Collection. Mechanical ventilation was defined as receipt of at least an hour of continuous ventilatory support or having procedure codes (classified using the 7th edition of the Australian Classification of Health Interventions) for airway management, invasive or non-invasive ventilatory support (eTable 1).

#### Model specification and estimation

As part of model development, a stepwise backward selection approach using Akaike Information criterion (AIC) was performed. Stepwise backward selection using AIC is a widely used criterion to assess model goodness of fit and parsimony^23,24^. We assessed the subsequent model improvement through a decrease in the AIC criterion after including all variables in the minimal sufficient adjustment sets plus additional variables not included in our DAG. All variables removed were individually reinserted into the model and reassessed for any model improvement.

#### Predictive performance and model validation

The ability of the variables to predict RSV-positivity in the source population was determined by a tenfold cross-validated area under the receiver operating characteristic (AUROC) curve. We constructed Receiver Operator Characteristic (ROC) by plotting the true positive rate (sensitivity) against false positive rate (1-specificity) at various levels of the marker. In k-fold cross validation, the dataset is randomly divided into k approximately equally sized subsamples (or folds)^25^. At each iteration, one-fold is retained as the validation data for testing the model and estimating the Area Under Curve (AUC), while the remaining k − 1 folds are used as training data for model estimation. This process is repeated k times, with each of the k folds used once as the validation data. The ‘*cvauroc’* command in Stata was employed to derive and average the AUCs corresponding to each fold (here k = 10) and bootstrapping the cross-validated AUC was used to obtain statistical inference and bias corrected 95% confidence intervals (CIs). K-fold cross validation avoids the optimistic estimates of predictive performance known to exist when the full dataset is used for assessing model specification and prediction^25^. To explore diagnostic accuracy of the models, sensitivity, specificity, positive predictive values (PPV) and negative predictive values (NPV) were generated. We determined these characteristics at different false positive rates [eTable 3]. However, we chose a 0.5 probability threshold as the cut-off, which maximises sensitivity and specificity of the model. Furthermore, we used a ‘calibration belt’ to evaluate the calibration of our predictive model. The ‘calibration belt’ is a plot depicting the relationship between the model’s fit probabilities and the observed proportions of the response across all ranges of risk, which reflects the reliability or degree of bias of the model^26^. In addition, reliability was tested by the Hosmer–Lemeshow goodness-of-fit test^27^. As a sensitivity analysis, we repeated the same procedure for developing the prediction model by method of testing to see if predictors of RSV-positivity differ between PCR or immunofluorescence (IF) detection methods (eFigs. 8 and 9).

All data were analysed using STATA v.16.0^28^. We used DAGitty v2.3 to produce the DAG^29^. We conducted a complete case analysis excluding records from the analysis with missing values for one or more of the predictor variables. We calculated incidence rates of predicted RSV-positivity using survival analysis techniques, allowing for multiple hospital admissions per person. We used person-time-at-risk as the denominator (calculated from date of birth until first date of hospital admission). All children were censored at the end of the study period or date of death, whichever was the earlier. Incidence rates were reported per 1000 child-years with associated 95% confidence intervals (CIs) by age group, year of hospital admission, admission season and birth month. Finally, we plotted predicted RSV rates by calendar week throughout the study period.

#### Estimating burden of RSV

After running each logistic regression model, we estimated a predicted probability of RSV-positivity and applied that to all hospital admissions in children aged less than 5 years during the study period given all non-missing variables in the model. All admissions in children aged less than 5 years during the study period with a predicted probability threshold of 0.5 were classified as an RSV-associated admission (hereafter referred to as predicted RSV). Finally, we estimated the under-ascertainment fraction of RSV rates, computed as rate differences between laboratory-confirmed RSV and predicted RSV.

#### Transparency of reporting

The Transparent Reporting of a multivariable prediction model for Individual Prognosis Or Diagnosis (TRIPOD) statement was followed for this study (eTable 4)^30^. The TRIPOD statement provides a framework for the full and clear reporting of a prediction model study, such that risk of bias and potential usefulness can be adequately assessed.

#### Ethics statement

The authors assert that all methods were carried out in accordance with relevant guidelines and regulations. Ethical approvals were obtained from the WA Department of Health Human Research Ethics Committee and the WA Aboriginal Health Ethics Committee. As the study utilised de-identified linked administrative data, a waiver of informed consent was granted by the WA Department of Health Human Research Ethics Committee.

## Results

### Cohort description

From the birth cohort of 321,825 hospitalised children under the age of 5 years at the time of hospital admission, 37,784 were tested for RSV (11.7%). Of these 8,471 (22.4%) were infants aged < 3 months and 5,768 (15.3%) were Aboriginal. From all hospitalised children tested for RSV, laboratory confirmation was determined in 22.8% (n = 8,604 episodes). RSV positivity was more common in children that were younger, non-Aboriginal, male, had mothers with average SES, lived in a metropolitan residence and had hospital admission in the Australian winter months between June and August. One in three (2,594 admissions) of RSV positive admissions were among infants aged < 3 months. Approximately three quarters of all laboratory-confirmed RSV-positive children had a discharge diagnosis of bronchiolitis (Table 1).

**Table 1:** Characteristics of laboratory-confirmed RSV-positive admissions used to generate the RSV prediction model, 2000–2012.

Characteristics		Total admissions N (%)	RSV-positive N (%)	RSV positivity rate (%)
		N = 37,784	N = 8,604	22.8
Age in months	< 3 months	8,471 (22.4)	2,594 (30.1)	31
	3–< 6 months	5,077 (13.4)	1,614 (18.8)	32
	6–< 12 months	7,641 (20.2)	1,718 (20.0)	22
	12–< 24 months	8,804 (23.3)	1,611 (18.7)	18
	24–< 36 months	3,773 (10.0)	599 (7.0)	16
	36- < 60 months	4,018 (10.6)	468 (5.4)	12
Admission season	Summer	5,298 (14.0)	301 (3.5)	6
	Autumn	6,735 (17.8)	967 (11.2)	14
	Winter	14,950 (39.6)	5,834 (67.8)	39
	Spring	10,801 (28.6)	1,502 (17.5)	14
Baby Aboriginal status	Non-Aboriginal	32,016 (84.7)	7,301 (84.9)	23
	Aboriginal	5,768 (15.3)	1,303 (15.1)	23
Delivery route	Vaginal	20,177 (53.4)	4,749 (55.2)	24
	Instrumental	3,698 (9.8)	756 (8.8)	20
	Elective Caesarean	6,558 (17.4)	1,566 (18.2)	24
	Emergency caesarean	7,337 (19.4)	1,529 (17.8)	21
Gender	Female	16,040 (42.5)	3,758 (43.7)	23
	Male	21,744 (57.5)	4,846 (56.3)	22
Maternal age	< 20	3,187 (8.4)	742 (8.6)	23
	20–24	7,603 (20.1)	1,773 (20.6)	23
	25–29	10,409 (27.6)	2,377 (27.6)	23
	30–34	10,262 (27.2)	2,316 (26.9)	23
	≥ 35	6,309 (16.7)	1,392 (16.2)	22
Smoking during pregnancy	Yes	9,141 (25.1)	2,208 (26.6)	24
Gestational age	< 32 weeks	1599 (4.2)	261 (3.0)	16
	32–36 weeks	6530 (17.3)	1516 (17.6)	23
	> 36 weeks	29,641 (78.4)	6823 (79.3)	23
SEIFA score at birth	0–10% (most deprived)	4,634 (13.2)	1,123 (14.1)	24
	11–25%	6,918 (19.7)	1,611 (20.2)	23
	26–75%	16,876 (48.0)	3,772 (47.3)	22
	76–90%	4,515 (12.8)	986 (12.4)	22
	91–100% (least deprived)	2,231 (6.3)	479 (6.0)	21
Number of siblings	0	8,943 (23.7)	1,720 (20.0)	19
	1	11,122 (29.4)	2,616 (30.4)	24
	2 or more	17,705 (46.9)	4,264 (49.6)	24
Maternal asthma		5,561 (14.7)	1,248 (14.5)	22
Mention of bronchiolitis in any of the diagnoses		11,865 (31.4)	6,245 (72.6)	53
Mention of pneumonia in any of the diagnoses		3,730 (9.9)	1,024 (11.9)	27
Mention of unspecified ALRI in any of the diagnoses		2,542 (6.7)	493 (5.7)	19
Any URTI in episode (Burgner categories)		6,450 (17.1)	891 (10.4)	14
Mention of influenza in any of the diagnoses		1,385 (3.7)	39 (0.5)	3
Mention of bronchitis in any of the diagnoses		185 (0.5)	57 (0.7)	31
Mention of asthma in any of the diagnoses		2,364 (6.3)	310 (3.6)	13
Mention of whooping cough in any of the diagnoses		307 (0.8)	23 (0.3)	7
Diagnostic code for other infections^a^		5,216 (13.8)	263 (3.1)	5
Remoteness	Metro	29,921 (79.3)	6,571 (76.4)	22
	Rural	4,100 (10.9)	1,068 (12.4)	26
	Remote	3,727 (9.9)	957 (11.1)	26
Length of hospital stay	1 day	2,357 (6.2)	255 (3.0)	11
	2 days	8,908 (23.6)	1,454 (16.9)	16
	3 or more days	26,519 (70.2)	6,895 (80.1)	26
ICU admission		2,976 (7.9)	492 (5.7)	17
Mechanical ventilation		1,122 (3.0)	213 (2.5)	19
Season of birth	Summer	8,699 (23.0)	1,813 (21.1)	21
	Autumn	10,758 (28.5)	2,883 (33.5)	27
	Winter	9,774 (25.9)	2,380 (27.7)	24
	Spring	8,553 (22.6)	1,528 (17.8)	18

*RSV* respiratory syncytial virus, *ALRI* acute lower respiratory infections, *URTI* upper respiratory tract infections, *ICU* intensive care unit.

^a^Other infections include unspecified viral illness, Convulsions, fever and cough, and breathing abnormalities.

### Predicting RSV positivity

The variables included in the final logistic regression prediction model were child age, gender, delivery route, admission season, Aboriginal status, maternal age, smoking, SES, numbers of siblings, prematurity, admission year, birth year, length of hospital stay, admission to ICU, mechanical ventilation use during admission, maternal history of asthma, any diagnosis code for ALRI and diagnosis code for other infections including unspecified viral illness. To account for seasonality and climatic conditions, we also included season of birth and geographical region of residence respectively (eTable 1). The predictive equation of the final model is presented in (eFig. 2).

In the multivariable analysis, there was a higher odd of RSV-positivity in those aged < 3 months (Adjusted odds ratio (AOR) = 1.91, 95% CI (1.27–2.87)), and children who were not Aboriginal (AOR = 1.44. 95% CI (1.29–1.61)). Children with a primary or secondary diagnosis of acute bronchiolitis had 16-fold increased odds of RSV-positivity (AOR = 16.8, 95% CI (15.3–18.5)). RSV-positivity was also significantly associated with a diagnosis of any pneumonia, unspecified ALRI and bronchitis. Similarly, children born in remote or rural areas, who had a long hospital stay (three or more days) and who required mechanical ventilation during admission had an increased odds of RSV positivity.

Conversely, prematurity, low SES and maternal history of asthma were associated with reduced odds of RSV-positivity (eTable 2).

**Figure 2:**
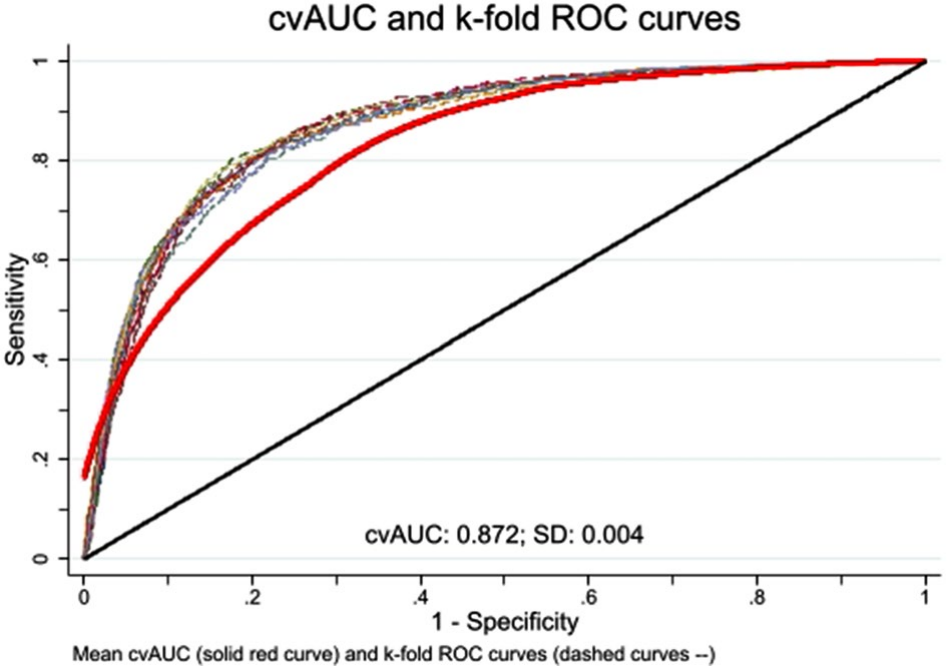
Tenfold cross-validated receiver operating characteristic ROC curves for RSV-associated admissions. Solid red curve = bias corrected cross-validated AUC, dashed curves = k-fold ROC curves.

The tenfold cross-validated model showed accurate and robust performance of the prediction model (AUROC = 0.87, 95% CI 0.86 to 0.88), reflecting excellent ability of the model to predict RSV-positivity (Fig. 2). The sensitivity and specificity of the final model were 58.4% (95% CI 57.3–59.6%), and 92.2% (95% CI 91.8–92.5%) respectively. The model had a PPV of 68.6% (95% CI 67.5–69.7%) and NPV of 88.3% (95% CI 87.9–88.7%). Additionally, the calibration belt demonstrated that our prediction model is well calibrated (eFig. 3). The overall goodness of fit of the model was satisfactory, as indicated by a nonsignificant Hosmer–Lemeshow test (*P* = 0.66). Our sensitivity analysis suggested that the prediction model performance slightly differed by laboratory detection method (immune-fluorescence vs PCR) (eFigs. 8 and 9).

### Laboratory-confirmed and predicted RSV rates by age at admission

The predicted incidence rates of RSV were higher in the younger age groups with the highest admission rates among infants aged less than 3 months. Our model predicted RSV-related admissions for children aged less than 3 months to be 43.7/1000 child-years (95% CI 42.1–45.4) compared with 31.7/1000 child-years (95% CI 30.3–33.1) from laboratory-confirmed RSV admissions. Similarly, the rate of predicted RSV admissions ranged from 5/1000 child-years for 12–24 months to 28/1000 child-years for 3–6 months of age. We estimated that the average annual RSV associated hospitalisation rates were 404 per 1000 children for < 3 months, 499 per 1000 children and 302 per 1000 children for 3–6 months and 6–11 months respectively, which is equivalent to under-ascertainment fractions of 32% for < 3 months, 57% for 3–6 months and 35% for 6–11 months of total admissions respectively (Table 2).

**Table 2:** Incidence rates of laboratory-confirmed and predicted RSV positivity by age at hospital admission, 2000–2012.

Age at admission	Laboratory-confirmed RSV		Predicted RSV		Under-ascertainment	
	Num	Rate (95% CI)	Num	Rate (95% CI)	Rate^a^	(%)^b^
0–27 days	625	21.3 (19.7–23.0)	704	24.0 (22.3–25.8)	2.7	13
28 days– < 3 months	1969	31.7 (30.3–33.1)	2716	43.7 (42.1–45.4)	12	38
< 3 months	2594	28.4 (27.2–29.5)	3420	37.4 (36.2–38.7)	9	32
3–< 6 months	1614	17.8 (16.9–18.7)	2531	27.9 (26.8–29.0)	10.1	57
6–< 12 months	1718	9.5 (9.1–10.0)	2311	12.8 (12.3–13.4)	3.3	35
12–< 24 months	1612	4.54 (4.3–4.8)	1873	5.3 (5.0–5.5)	0.7	16

All rates presented in this table are per 1000 child-years.

^a^Rate differences (per 1000 child-years) between lab-confirmed and predicted RSV.

^b^Percentage differences between rates of laboratory-confirmed RSV admissions & predicted RSV admissions computed using ((predicted RSV-laboratory confirmed RSV))/(laboratory confrimed RSV)*100

A total of 76% of our predicted RSV-associated admissions were in infants aged less than 12 months, accounting for 39% of the estimated under-ascertainment. For infants aged less than 12 months, the predicted RSV-associated admissions peaked during the winter season (n = 6859, 204/1000 child years [95% CI 199.1, 208.7)]) and in infants born in months of April–June (Table 3). Similarly, for children 12–24 months of age the predicted RSV admissions peaked during the winter season and in children born in March–May (Table 4).

**Table 3:** Incidence rates of laboratory-confirmed and predicted RSV positivity by selected patient characteristics among children aged < 12 months, 2000–2012.

0–12 months					
	Laboratory-confirmed RSV		Predicted RSV		
	Num	Rate/1000 child-years	Num	Rate/1000 child-years	Rate difference/1000 child-years
		(95% CI)		(95% CI)	
**Admission year**					
2000	461	18.3 (16.7, 20.0)	799	31.7 (29.6, 33.9)	13.4
2001	390	15.7 (14.2, 17.4)	654	26.4 (24.4, 28.5)	10.6
2002	600	24.3 (22.4, 26.3)	760	30.8 (28.7, 33.1)	6.5
2003	352	14.4 (13.0, 16.0)	554	22.7 (20.9, 24.7)	8.3
2004	475	19.0 (17.4, 20.8)	678	27.1 (25.2, 29.2)	8.12
2005	449	17.4 (15.9, 19.1)	718	27.8 (25.8, 29.9)	10.4
2006	521	18.9 (17.4, 20.6)	721	26.2 (24.3, 28.2)	7.3
2007	329	11.3 (10.1, 12.5)	486	16.6 (15.2, 18.2)	5.4
2008	513	16.9 (15.6, 18.5)	712	23.6 (21.9, 25.4)	6.6
2009	391	12.8 (11.6, 14.2)	476	15.6 (14.3, 17.1)	2.8
2010	497	15.9 (14.6, 17.4)	589	18.9 (17.4, 20.5)	2.9
2011	432	13.8 (12.5, 15.1)	457	14.6 (13.3, 15.9)	0.8
2012	516	15.9 (14.6, 17.3)	658	20.3 (18.8, 21.9)	4.4
**Admission season**					
Summer	193	7.9 (6.9, 9.2)	21	0.8 (0.6, 1.3)	− 7.1
Autumn	626	23.4 (21.6, 25.3)	612	22.9 (21.1, 24.7)	− 0.5
Winter	4008	119.1 (115.5, 122.9)	6859	203.8 (199.1, 208.7)	84.7
Spring	1099	33.8 (31.9, 35.9)	770	23.7 (22.1, 25.5)	− 10.1
**Birth month**					
Jan	424	42.4 (38.6, 46.7)	724	59.1 (54.5, 64.0)	16.62
Feb	446	46.9 (42.7, 51.4)	821	73.9 (68.6, 79.6)	27.02
Mar	599	57.8 (53.4, 62.7)	1087	87.9 (82.4, 93.8)	30.13
Apr	646	64.9 (60.2, 70.2)	1251	102.1 (96.0, 108.6)	37.11
May	805	78.9 (73.7, 84.6)	1395	112.6 (106.3, 119.3)	33.64
Jun	754	79.5 (74.1, 85.4)	1196	103.2 (96.9, 109.8)	23.63
Jul	542	55.8 (51.3, 60.7)	896	70.5 (65.4, 75.9)	14.64
Aug	410	41.9 (38.0, 46.2)	576	45.1 (41.1, 49.5)	3.17
Sep	335	34.2 (30.7, 38.1)	511	41.9 (38.1, 46.2)	7.77
Oct	292	29.8 (26.6, 33.5)	512	42.9 (39.0, 47.3)	13.09
Nov	325	35.3 (31.7, 39.4)	558	49.3 (44.9, 54.0)	13.92
Dec	348	37.3 (33.6, 41.5)	608	54.5 (49.9, 59.4)	17.16

**Table 4:** Incidence rates of laboratory-confirmed and predicted RSV positivity by selected patient characteristics among children aged 12–24 months, 2000–2012.

12–24 months					
	Laboratory-confirmed RSV		Predicted RSV		
	Num	Rate /1000 child-years	Num	Rate /1000 child-years	Rate difference /1000 child-years
		(95% CI)		(95% CI)	
**Admission year**					
2000	143	5.6 (4.7, 6.6)	166	6.5 (5.6, 7.6)	0.89
2001	85	3.4 (2.7, 4.2)	128	5.1 (4.3, 6.0)	1.71
2002	153	6.2 (5.3, 7.2)	154	6.2 (5.3, 7.3)	0.04
2003	91	3.7 (3, 4.5)	135	5.5 (4.6, 6.5)	1.78
2004	124	5.1 (4.3, 6.1)	137	5.6 (4.8, 6.7)	0.53
2005	93	3.7 (3.0, 4.6)	138	5.5 (4.7, 6.5)	1.80
2006	123	4.8 (4.0, 5.7)	148	5.7 (4.9, 6.7)	0.97
2007	88	3.2 (2.6, 3.9)	159	5.8 (4.9, 6.8)	2.58
2008	131	4.5 (3.8, 5.3)	188	6.4 (5.6, 7.4)	1.95
2009	120	3.9 (3.3, 4.8)	121	4.0 (3.4, 4.8)	0.04
2010	157	5.2 (4.4, 6.0)	169	5.6 (4.8, 6.5)	0.39
2011	168	5.4 (4.7, 6.3)	126	4.1 (3.4, 4.8)	− 1.34
2012	136	4.3 (3.7, 5.1)	104	3.3 (2.7, 4.0)	− 1.02
**Admission season**					
Summer	70	3.5 (2.8, 4.4)	12	0.6 (0.3, 1.1)	− 2.9
Autumn	216	9.9 (8.6, 11.3)	201	9.2 (8.0, 10.5)	− 0.7
Winter	1083	41.8 (39.4, 44.4)	1496	57.8 (54.9, 60.7)	15.9
Spring	243	9.2 (8.07, 10.4)	164	6.2 (5.3, 7.2)	− 2.9
**Birth month**					
Jan	124	15.2 (12.8, 18.1)	590	59.1 (54.5, 64.0)	43.8
Feb	125	16.3 (13.6, 19.4)	703	73.9 (68.6, 79.6)	57.6
Mar	158	19.0 (16.3, 22.2)	911	88.0 (82.5, 93.8)	69.0
Apr	167	20.8 (17.9, 24.2)	1015	102.1 (96.0, 108.6)	81.3
May	165	20.1 (17.2, 23.4)	1148	112.6 (106.3, 119.3)	92.5
Jun	165	21.6 (18.6, 25.2)	978	103.2 (96.9, 109.8)	81.5
Jul	137	17.6 (14.9, 20.8)	684	70.5 (65.4, 75.9)	52.9
Aug	129	16.5 (13.8, 19.5)	441	45.1 (41.1, 49.5)	28.6
Sep	127	16.3 (13.7, 19.4)	411	42.0 (38.1, 46.3)	25.7
Oct	105	13.4 (11.0, 16.2)	420	42.9 (39.0, 47.3)	29.6
Nov	101	13.5 (11.1, 16.5)	453	49.3 (44.9, 54.0)	35.7
Dec	109	14.4 (12.0, 17.4)	508	54.5 (49.9, 59.4)	40.0

### RSV rates by calendar week

**Figure 3:**
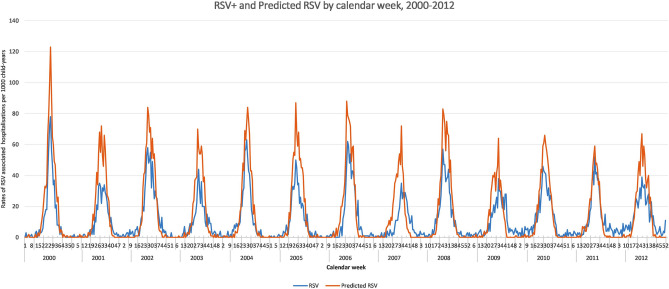
Laboratory- confirmed hospitalised RSV positive and predicted RSV cases by calendar week for children < 2 years, 2000–2012.

Rates of predicted RSV admissions for children aged less than 2 years showed a seasonal pattern. The peak in predicted RSV-associated admissions for this age group were observed during week 26–29 each year between 2000 to 2012, with exception of year 2007 and 2009 with a peak observed during week 34 (66/1000 child-years) and week 32 (55.9/1000 child-years), respectively. We observed a similar pattern to the peak in admissions for laboratory-confirmed RSV admissions during the study period (Fig. 3).

## Discussion

RSV vaccine development has gained substantial attention globally, with the WHO identifying global RSV disease burden estimates as a global priority^31,32^. Our study aimed to improve the estimates of RSV incidence which are needed to help advocate for vaccine programs and provide essential baseline data to evaluate vaccine impact studies. We have developed a population-based prediction model to better estimate the true burden of RSV associated hospitalisations in children younger than 5 years in WA with robust performance. Our prediction model was based on readily available patient characteristics and may be useful for identifying hospitalised children likely to test positive for RSV.

Using population-based hospital and laboratory data, our results corroborated previous findings of the large burden of RSV-associated hospital admissions in children^3,4,7^, and our prediction model suggests that we underestimate this burden by more than 30%. Our results also indicate that the RSV burden is age-specific, with significant seasonal variation. Over the study period, which spanned more than a decade, the burden of predicted RSV-associated hospital admissions was substantial, with an average annual estimated admission rate of 404 per 1000 children for < 3 months and 338 per 1000 children for under 2 years old respectively. This is consistent with our previous results^3,4^ and with findings from other studies^33,34^. The peaks in RSV-associated admissions were observed in the winter season, as well as in children born in April, May and June in both < 12 months and 12–24 months age groups. Given the higher burden of RSV-associated hospitalisation among the young infant age group, future vaccine programmes could target them as priority beneficiaries. There are recent suggestions of targeting infants born around the start of RSV season for a possible seasonal vaccination strategy^35^.

Our analysis confirmed that children who are younger at admission (< 3 months) are at increased risk of RSV positivity, which is consistent with other studies^7,36^. In addition to younger age, our study also identified additional important predictors of RSV positivity, including length of hospital stay, primary or secondary diagnosis of acute bronchiolitis, pneumonia, bronchitis and unspecified ALRIs. A primary or secondary diagnosis of acute bronchiolitis was the strongest predictor of RSV positivity. A diagnosis of bronchitis was the second most important predictor, followed by pneumonia. A previous study in England similarly reported that infants with a diagnosis of bronchiolitis, unspecified LRTI or with an RSV-specific code had higher odds of RSV-positivity^7^.

The risk of hospitalisation was higher for non-Aboriginal children and children from a rural or remote region at birth. Similarly, the highest rates of RSV were found in remote and rural regions compared to metropolitan areas. These difference could be attributed to a combination of socio economic disadvantages (such as household crowding) and access to health care^37,38^. Interestingly, low SES, prematurity and family history of asthma were associated with lower odds of RSV positivity. Similar findings were reported in previous studies^7,39^. Nonetheless, findings reported elsewhere have suggested that the majority of cases with RSV do not have any underlying co-morbidity^40^.

Our evaluation using tenfold cross validation showed an excellent performance and was well calibrated, with AUROC of 0.87^27^. By comparison, in a recent study predicting RSV associated admissions in England reported AUROC of 0.9, our predictive model has higher specificity and NPV but lower sensitivity and PPV compared to that model which focused on infants under the age of 12 months^7^. Our prediction study differs from others with respect to either the domain or population studied. Other studies focused on RSV hospitalisation in young children^7,41^, premature infants^42,43^, or non-hospitalised RSV among healthy term infants^44^.

Our prediction model showed a good fit to the seasonality and age distribution of RSV-associated hospitalisation. However, the model slightly underestimated the RSV-associated admissions for older children. This could be due to non-inclusion of additional risk factors, or lifestyle factors beyond the perinatal period, where we had limited data to base our prediction model on. Our prediction model was based on more than a decade of population-based hospitalisation data linked with laboratory and socio-demographic records, which is a key strength of our study. Our model included a comprehensive list of maternal, infant, and perinatal predictors which were not included in previous similar RSV prediction studies^7,41,45^. We also employed a DAG as an efficient strategy to improve predictor selection in the prediction modelling. We believe the definition of ALRIs, and other infections included as risk factors in our prediction model increases the likelihood of positive detection of a respiratory virus in hospital admissions. We also employed a k-fold cross validation, an internal validation method that takes over-optimism into account far better than conventional data splitting^46^.

Our study has some limitations. Firstly, our results are based on hospitalisation data linked to laboratory records with specimen collection within 48 h of hospital admission. Therefore, we are limiting our prediction model to more severe RSV cases associated with hospitalisation and therefore our model does not estimate the broader community incidence of RSV. Secondly, our analysis only included linked data in the years between 2000–2012, and more recent trends in hospital admissions are not reflected in our results. However, we are confident our model can be applied to more contemporary data and believe that the majority of the known perinatal and environmental predictors are not likely to change over time. Our study did not include other potential predictors such as breastfeeding, immunodeficiencies, day care attendance, and environmental factors, as these factors are not routinely collected in databases available for linkage. However, we do not expect a major underestimation of our prediction model as these variables are not known to be strong predictors of RSV associated admissions^7,44^. Additionally, even though the testing detection method would not impact the propensity to get tested, our sensitivity analysis suggested that the prediction model performance slightly different by laboratory detection method.

The population-based linkage of routine laboratory and hospitalisation data allowed us to develop a predictive model with excellent predictive performance to identify RSV associated hospitalisation in WA. Applying the model to all hospitalised children aged less than 5 years (irrespective of respiratory infection diagnosis) during the study period enabled us to estimate the true RSV burden in hospitalised children in the state.

Further research is needed that takes into consideration emergency department and community RSV-associated admissions, preferably including recent data, as well as other potential predictors. A development of a simple risk score, and further external validation of the model in other populations must also be considered for possible future clinical use of the prediction model.

## Conclusion

We have successfully developed a prediction model using population-based data to estimate the true burden of RSV in hospitalised children in WA with good predictive performance and internal validation. Findings from our study indicate that the true burden of RSV is up to 30–57% higher than figures based solely on laboratory detection data in young children. These estimates can now be used as input parameters in dynamic transmission models to better predict the impact of prevention measures including maternal vaccination^47^.

### Supplementary Information

Supplementary Information.
