# Author Correction: Developing a prediction model to estimate the true burden of respiratory syncytial virus (RSV) in hospitalised children in Western Australia

**DOI:** 10.1038/s41598-024-52791-0

**Published:** 2024-01-29

**Authors:** Amanuel Tesfay Gebremedhin, Alexandra B. Hogan, Christopher C. Blyth, Kathryn Glass, Hannah C. Moore

**Affiliations:** 1 Wesfarmers Centre of Vaccines and Infectious Diseases, Telethon Kids Institute, University of Western Australia, Perth, 6872 Australia; 2 MRC Centre for Global Infectious Disease Analysis, School of Public Health, Imperial College London, London, UK; 3 School of Medicine, The University of Western Australia, Perth, WA Australia; 4 Department of Infectious Diseases, Perth Children’s Hospital, Perth, WA Australia; 5 PathWest Laboratory Medicine, QEII Medical Centre, Nedlands, Perth, WA Australia; 6 Research School of Population Health, Australian National University, Canberra, Australia

Correction to: *Scientific Reports* 10.1038/s41598-021-04080-3, published online 10 January 2022

The original version of this Article contained errors in Table 1, where the values for “RSV-positive N (%)” under the Gestational age variable were interchanged. As a result, the values for “Total admissions N (%)” and “RSV positivity rate (%)” were incorrect. The correct and incorrect values appear below.

Incorrect:

**Table:** 

Characteristics		Total admissions N (%)	RSV-positive N (%)	RSV positivity rate (%)
Gestational age	< 32 weeks	27,322 (74.9)	261 (3.0)	22
	32–36 weeks	1,599 (4.2)	1,516 (17.6)	16
	> 36 weeks	6,530 (17.3)	6,823 (79.3)	23

Correct:

**Table:** 

Characteristics		Total admissions N (%)	RSV-positive N (%)	RSV positivity rate (%)
Gestational age	< 32 weeks	1599 (4.2)	261 (3.0)	16
	32–36 weeks	6530 (17.3)	1516 (17.6)	23
	> 36 weeks	29,641 (78.4)	6823 (79.3)	23

The original Article has been corrected.

