# Supplementary material for: Developing a prediction model to estimate the true burden of respiratory syncytial virus (RSV) in hospitalised children in Western Australia
Source: Sci Rep. 2022 Jan 10;12:332. doi: 10.1038/s41598-021-04080-3 (PMC8748465; doi:10.1038/s41598-021-04080-3)
Supplement: Supplementary file 1 — Supplementary Information. [file 41598_2021_4080_MOESM1_ESM.pdf]

Supplementary files

Developing a prediction model to estimate the true burden of respiratory syncytial virus (RSV) in hospitalised children in Western Australia

Authors: Amanuel Tesfay Gebremedhin[1,*], Alexandra B Hogan[2], Christopher C Blyth[1,3,4,5], Kathryn Glass[6], Hannah C Moore[1]

Affiliations:

[1]Wesfarmers Centre of Vaccines and Infectious Diseases, Telethon Kids Institute, University of Western Australia, Perth, Australia

[2]MRC Centre for Global Infectious Disease Analysis, School of Public Health, Imperial College London, UK

[3]School of Medicine, The University of Western Australia, Perth, WA, Australia,

[4]Department of Infectious Diseases, Perth Children's Hospital, Perth, Western Australia, Australia,

[5]PathWest Laboratory Medicine, QEII Medical Centre, Nedlands, Perth, WA, Australia

[6]Research School of Population Health, Australian National University, Canberra

[*]**Corresponding Author**

Amanuel Tesfay Gebremedhin (PhD)

Wesfarmers Centre of Vaccines and Infectious Diseases,

Telethon Kids Institute,

The University of Western Australia,

Western Australia, 6872, Australia

Email: Amanuel.gebremedhin@telethonkids.org.au

Phone: 0451584142

List of figures

eFigure 1. Directed Acyclic Graphs (DAGs) for the predictors of RSV positivity 3
eFigure 2. Predictive equation 4
eFigure 3. Calibration belt of prediction model 5
eFigure 4. Number of laboratory confirmed RSV-positive admissions and the number predicted RSV by the final model, by age at admission 13
eFigure 5. Number of laboratory confirmed RSV-positive admissions and the number predicted RSV by the final model, by age diagnosis at admission 14
eFigure 6. Number of laboratory confirmed RSV-positive admissions and the number predicted RSV by the final model, by age admission year . 15
eFigure 7. Number of laboratory confirmed RSV-positive admissions and the number predicted RSV by the final model, by age admission season 16
eFigure 8. Receiver-operator curve (ROC) and AUC score computed with 10-fold cross-validation for predicted RSV using immune-fluorescence (IF) test 17
eFigure 9. Receiver-operator curve (ROC) and AUC score computed with 10-fold cross-validation for predicted RSV using *PCR test* 18

List of tables

eTable 1. Diagnostic and procedure codes 6
eTable 2. Logistic regression of predictors of Laboratory confirmed RSV-positivity among hospitalized children aged <5 years in WA, 2000-2012 . 7
eTable 3. Model performance of the prediction model 10
eTable 4. The Transparent Reporting of a multivariable prediction model for Individual Prognosis Or Diagnosis (TRIPOD) statement 11

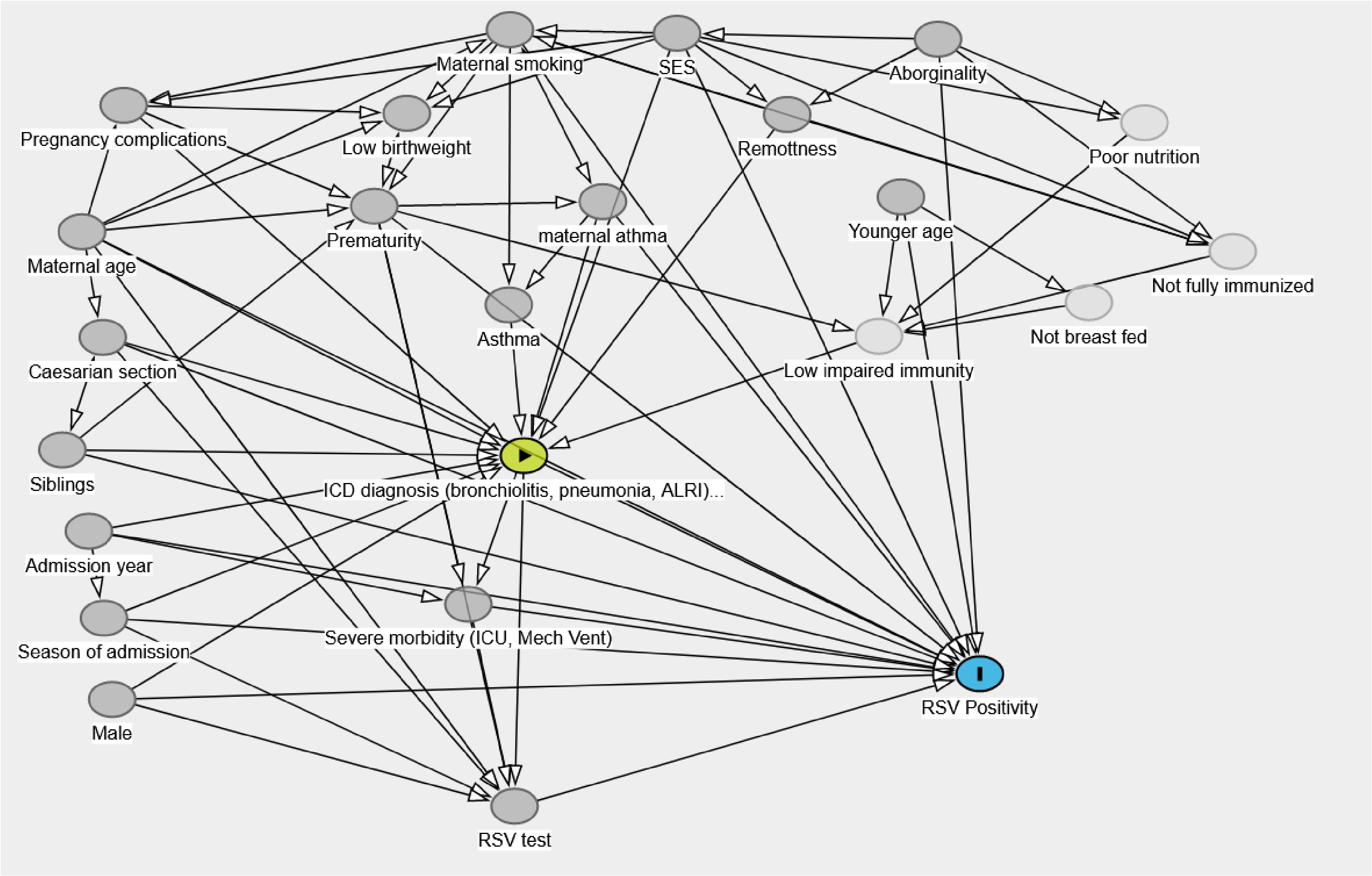

eFigure 1. Directed Acyclic Graphs (DAGs) for the predictors of RSV positivity

$log\left[\frac{p}{1-p}\right]$ =-1.499535 + 2.822659**any acute bronchiolitis* + 1.584008**any bronchitis* + 1.312106**any pneumonia* + 1.020578**any unspecified ALRI* + 0.3071243**any URTI* + 0.6494178 * *age admission[3 months]* + 0.3648039 **age admission[3-6 months]* - 0.1614355 **age admission[6-12 months]* + 0.2802494* *age admission[12-24 months*] + 0.4934716**age admission[24-36 months]* - 0.8410958**admission season [Autumn]* -0.4609775**admission season [winter]*- 0.5331982**admission season [Spring*] + 0.4521587**remote [residence]* + 0.3279418**rural [residence*] + 0.3675772**Non-indigenous* + 0.3499608**any asthma* - 0.2324519**any other infections*- 0.1549112* *any whooping cough* - 0.0422594**delivery route [Instrumental]* + 0.003504**delivery route [Elective Caesarean]* - 0.0542601**delivery route [Emergency caesarean]* - 0.0997978**male* + 0.0648211**mother age [20-24*] + 0.0486902**mother age [25-29*] + 0.0379241**mother age [30-34]* + 0.09721**mother age [≥35]* - 0.0512**mother smoke* - 0.0841153**SEIFA score [0-10%]* - 0.086608**SEIFA* score [11-25%] - 0.0768222**SEIFA score [26-75%]* - 0.0688228**SEIFA score [76-90%]* + 0.0625687 **number of siblings [1]* - 0.0298783**number of siblings [2]* - 0.1186202**number of siblings [3]* - 0.0483282**number of siblings [≥4]* - 0.0999879**mother asthma* +0.0101987**length of stay[2 days]*+0.4762351**length of stay[≥3 days]* + 0.0070008**mechanical ventilation* - 0.509525**ICU admission* - 0.0989403**birth season [Summer]* - 0.1051929**birth season [Autumn]* - 0.0972795**birth season [Winter]* - 0.0983472**birth year* + 0.1003071**admission year* - 0.1071373*$sin\left(\frac{2\pi t}{52}\right) - 1.499535 * cos\left(\frac{2\pi t}{52}\right)$

eFigure 2. Predictive equation

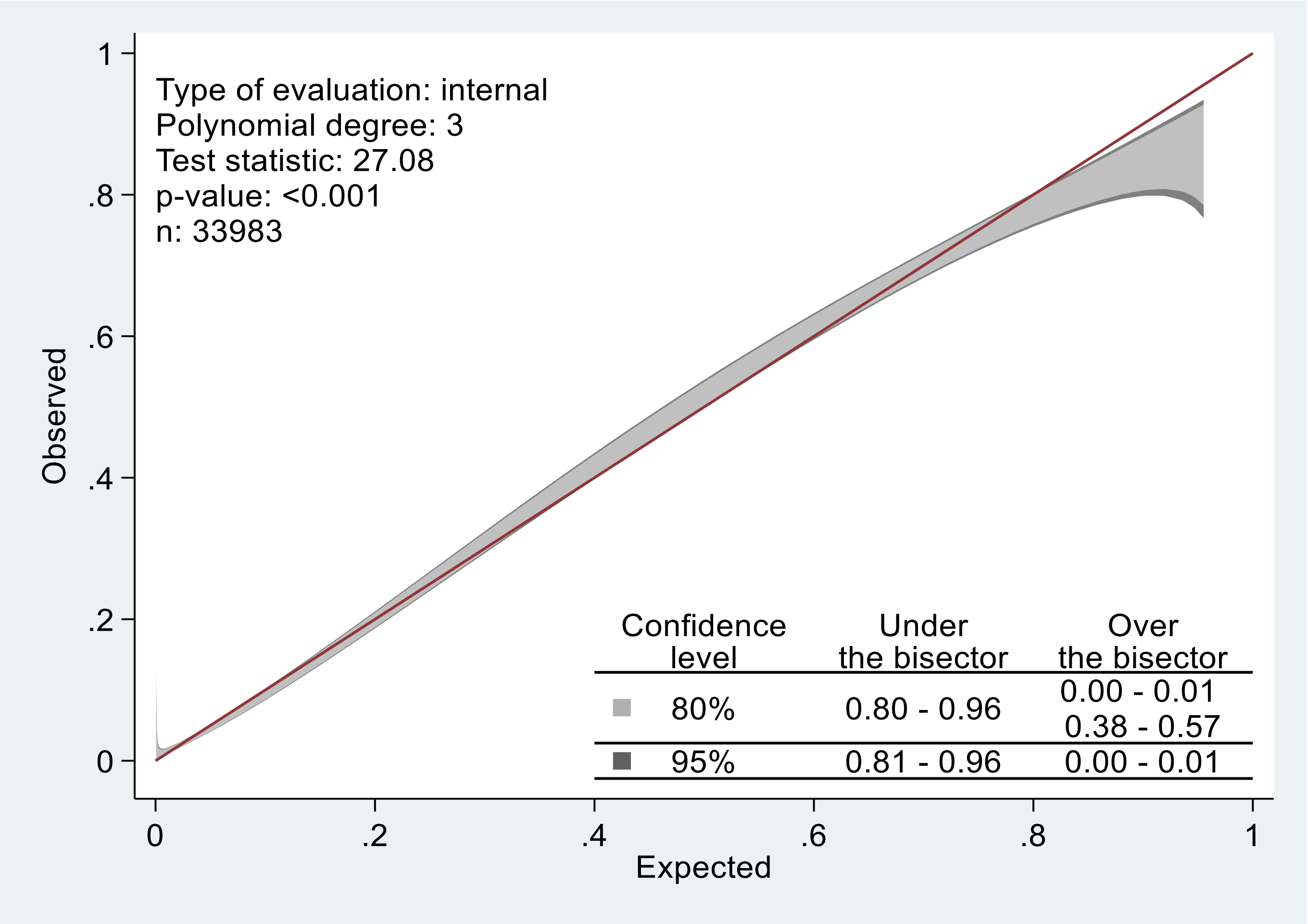

eFigure 3. Calibration belt of prediction model

A calibration plot, showing the agreement between the hospitalization risks predicted from our prediction model and the real observed RSV hospitalization. The ‘red line’ indicates the ideal situation of perfect agreement; while ‘the grey shaded’ area shows the calibration curve of our prediction rule.

eTable 1. Diagnostic and procedure codes

Description	Codes used	Type
**ALRI**		
Whooping cough	A37	Any diagnosis
Pneumonia	J12-18	Any diagnosis
Acute bronchiolitis	J21	Any diagnosis
Influenza due to identified virus	J09-J10	Any diagnosis
Unspecified ALRI	J22	Any diagnosis
Bronchitis	J20, J40	Any diagnosis
Asthma	J45, J46	Any diagnosis
**URTI**		
Diphtheria	A36.0 – A36.2	Any diagnosis
Otitis media and other ear conditions	B05.3, H65-H67, H68.0, H72, H73.0, H83.0, H92.1	Any diagnosis
Mastoiditis and related conditions	H70, H75.0	Any diagnosis
Acute upper respiratory infections (incl. pharyngitis)	J00-J04, J06	Any diagnosis
Epiglottitis	J05.1	Any diagnosis
Chronic sinusitis	J32	Any diagnosis
Other nose, tonsil or adenoid disorders	J34.0, J35.0, J36	Any diagnosis
**Diagnostic code for other infections**		
Viral infection of unspecified site	B34	Any diagnosis
Other infections	AXX, BXX unless included in other categories	Any diagnosis
Cystic fibrosis	E84	Any diagnosis
Other respiratory diseases	JXX unless included in other categories	Any diagnosis
Breathing abnormalities (incl. cough)	R05, R06	Any diagnosis
Convulsions (incl. febrile)	R56, P90	Any diagnosis
Fever	R50	Any diagnosis
Abnormal clinical signs, symptoms and laboratory findings	RXX unless included in other categories	Any diagnosis
**Mechanical ventilation**		
Airway management	22007-00, 22007-01, 22008-00, 22008-01, 90179-02, 92035-00, 92041-00	Procedures
Non-invasive ventilatory support	92209-XX	Procedures
Ventilatory support	13882-XX	Procedures

eTable 2. Logistic regression of predictors of Laboratory confirmed RSV-positivity among hospitalized children aged <5 years in WA, 2000-2012

Characteristics	AOR (95% CI)
**Age at admission**	
<3 months	1.91 (1.28, 2.87)
3-<6 months	1.44 (0.97, 2.13)
6-<12 months	0.85 (0.60, 1.21)
12-<24 months	1.32 (0.99, 1.77)
24-<36 months	1.64 (1.32, 2.04)
36-<60 months	(Reference)
**Season of admission**	
Summer (December–February)	(Reference)
Autumn (March–May)	0.43 (0.34, 0.55)
Winter (June–August)	0.63 (0.48, 0.83)
Spring (September–November)	0.59 (0.47, 0.74)
**Aboriginal status**	
Non-Aboriginal	1.44 (1.29, 1.61)
Aboriginal	(Reference)
**Mode of delivery**	
Vaginal	(Reference)
Instrumental	0.96 (0.85, 1.08)
Elective Caesarean	1.00 (0.92, 1.10)
Emergency caesarean	0.95 (0.86, 1.04)
**Gender**	
Female	(Reference)
Male	0.91 (0.85, 0.97)
**Maternal age**	
<20	(Reference)
20-24	1.07 (0.93, 1.23)
25-29	1.05 (0.91, 1.21)
30-34	1.04 (0.90, 1.20)
>=35	1.10 (0.94, 1.29)
**Mother smoked during pregnancy**	
Yes	0.95 (0.87, 1.03)
No	(Reference)
**Gestational age**	
<32 weeks	0.55 (0.45, 0.66)
32-36 weeks	0.88 (0.80, 0.96)
>36 weeks	(Reference)
**SEIFA at birth**	
0-10% (most disadvantaged)	0.92 (0.78, 1.09)
11-25%	0.92 (0.78, 1.07)
26-75%	0.93 (0.80, 1.07)

76-90%		0.93 (0.80, 1.10)
91-100% (most advantaged)		(Reference)
**Number of older siblings at birth**		
	0	(Reference)
	1	1.06 (0.96, 1.18)
	2	0.97 (0.87, 1.08)
	3	0.89 (0.78, 1.01)
	≥4	0.95 (0.84, 1.08)
**Mother asthma**		
No		(Reference)
Yes		0.90 (0.82, 0.99)
**Any acute bronchiolitis**		
No		(Reference)
Yes		16.82 (15.31, 18.49)
**Any pneumonia**		
No		(Reference)
Yes		3.71 (3.28, 4.20)
**Any unspecified ALRI**		
No		(Reference)
Yes		2.77 (2.41, 3.20)
**Other infections**		
No		(Reference)
Yes		0.79 (0.68, 0.93)
**URTI**		
No		(Reference)
Yes		1.36 (1.22, 1.52)
**Any influenza**		
No		(Reference)
Yes		0.17 (0.11, 0.24)
**Any bronchitis**		
No		(Reference)
Yes		4.87 (3.21, 7.40)
**Any asthma**		
No		(Reference)
Yes		1.42 (1.21, 1.67)
**Any whooping cough**		
No		(Reference)
Yes		0.86 (0.51, 1.43)
**Region at birth**		
Metropolitan		(Reference)
Rural		1.39 (1.24, 1.56)
Remote		1.57 (1.38, 1.80)
**Length of stay**		
1 d		(Reference)

2 d	1.01 (0.84, 1.21)
3 or more days	1.61 (1.36, 1.91)
**Mechanical ventilation**	
No	(Reference)
Yes	1.01 (0.80, 1.27)
**ICU admission**	
No	(Reference)
Yes	0.60 (0.52, 0.70)
**Season of birth**	
Spring	(Reference)
Summer	0.91 (0.81, 1.02)
Autumn	0.90 (0.80, 1.01)
Winter	0.91 (0.82, 1.01)
**Birth year** [a]	0.91 (0.82, 1.00)
**Admission year** [a]	1.11 (1.00, 1.22)
sin	0.90 (0.81, 1.00)
cos	0.22 (0.20, 0.26)

[a] modelled as continuous. Abbreviations: ALRI, Acute Lower Respiratory Infections; URTI, Upper Respiratory Tract Infections; ICU, Intensive Care Unit

eTable 3. Model performance of the prediction model

Predicted Probability	Sensitivity	Specificity	FP
0.01	99.31	14.57	85.43
0.1	93.07	56.85	43.15
0.2	83.16	76.6	23.4
0.3	75.05	83.82	16.18
0.4	66.93	88.19	11.81
**0.5**	**59.44**	**91.55**	**8.45**
0.6	47	94.64	5.36
0.7	20.06	98.09	1.57
0.8	2.73	99.67	0.33
0.9	0.62	99.95	0.05
0.96	0	100	0

eTable 4. The Transparent Reporting of a multivariable prediction model for Individual Prognosis Or Diagnosis (TRIPOD) statement

Section/Topic	Item		Checklist Item	Page
**Title and abstract**				
Title	1	D;V	Identify the study as developing and/or validating a multivariable prediction model, the target population, and the outcome to be predicted.	1
Abstract	2	D;V	Provide a summary of objectives, study design, setting, participants, sample size, predictors, outcome, statistical analysis, results, and conclusions.	2
**Introduction**				
Background and objectives	3a	D;V	Explain the medical context (including whether diagnostic or prognostic) and rationale for developing or validating the multivariable prediction model, including references to existing models.	3
	3b	D;V	Specify the objectives, including whether the study describes the development or validation of the model or both.	3-4
**Methods**				
Source of data	4a	D;V	Describe the study design or source of data (e.g., randomized trial, cohort, or registry data), separately for the development and validation data sets, if applicable.	4
	4b	D;V	Specify the key study dates, including start of accrual; end of accrual; and, if applicable, end of follow-up.	4-5
Participants	5a	D;V	Specify key elements of the study setting (e.g., primary care, secondary care, general population) including number and location of centres.	4-5
	5b	D;V	Describe eligibility criteria for participants.	4
	5c	D;V	Give details of treatments received, if relevant.	na
Outcome	6a	D;V	Clearly define the outcome that is predicted by the prediction model, including how and when assessed.	4-5
	6b	D;V	Report any actions to blind assessment of the outcome to be predicted.	4-5
Predictors	7a	D;V	Clearly define all predictors used in developing or validating the multivariable prediction model, including how and when they were measured.	6
	7b	D;V	Report any actions to blind assessment of predictors for the outcome and other predictors.	6
Sample size	8	D;V	Explain how the study size was arrived at.	4
Missing data	9	D;V	Describe how missing data were handled (e.g., complete-case analysis, single imputation, multiple imputation) with details of any imputation method.	8
Statistical analysis methods	10a	D	Describe how predictors were handled in the analyses.	6
	10b	D	Specify type of model, all model-building procedures (including any predictor selection), and method for internal validation.	7
	10c	V	For validation, describe how the predictions were calculated.	7
	10d	D;V	Specify all measures used to assess model performance and, if relevant, to compare multiple models.	7-8
	10e	V	Describe any model updating (e.g., recalibration) arising from the validation, if done.	8
Risk groups	11	D;V	Provide details on how risk groups were created, if done.	n.a
Development vs. validation	12	V	For validation, identify any differences from the development data in setting, eligibility criteria, outcome, and predictors.	

**Results**				
Participants	13a	D;V	Describe the flow of participants through the study, including the number of participants with and without the outcome and, if applicable, a summary of the follow-up time. A diagram may be helpful.	4
	13b	D;V	Describe the characteristics of the participants (basic demographics, clinical features, available predictors), including the number of participants with missing data for predictors and outcome.	9-10
	13c	V	For validation, show a comparison with the development data of the distribution of important variables (demographics, predictors and outcome).	n.a
Model development	14a	D	Specify the number of participants and outcome events in each analysis.	9
	14b	D	If done, report the unadjusted association between each candidate predictor and outcome.	n.a
Model specification	15a	D	Present the full prediction model to allow predictions for individuals (i.e., all regression coefficients, and model intercept or baseline survival at a given time point).	10
	15b	D	Explain how to the use the prediction model.	10
Model performance	16	D;V	Report performance measures (with CIs) for the prediction model.	10-11
Model-updating	17	V	If done, report the results from any model updating (i.e., model specification, model performance).	n.a
**Discussion**				
Limitations	18	D;V	Discuss any limitations of the study (such as nonrepresentative sample, few events per predictor, missing data).	13
Interpretation	19a	V	For validation, discuss the results with reference to performance in the development data, and any other validation data.	13-14
	19b	D;V	Give an overall interpretation of the results, considering objectives, limitations, results from similar studies, and other relevant evidence.	12-14
Implications	20	D;V	Discuss the potential clinical use of the model and implications for future research.	14
**Other information**				
Supplementary information	21	D;V	Provide information about the availability of supplementary resources, such as study protocol, Web calculator, and data sets.	
Funding	22	D;V	Give the source of funding and the role of the funders for the present study.	15

*Items relevant only to the development of a prediction model are denoted by D, items relating solely to a validation of a prediction model are denoted by V, and items relating to both are denoted D;V.

Additional figures for fit of final model – by age at admission, diagnosis, admission year and season.[1]

Our prediction model was a good fit for seasnality and age distribution of RSV-associated hospitalisation. However, the model slightly underestimated the RSV-associated admissions for older children. The model also over-predicted any diagnosis of acute bronchiolitis and underestimated the numbers of any pneumonia and ALRI diagnosis.

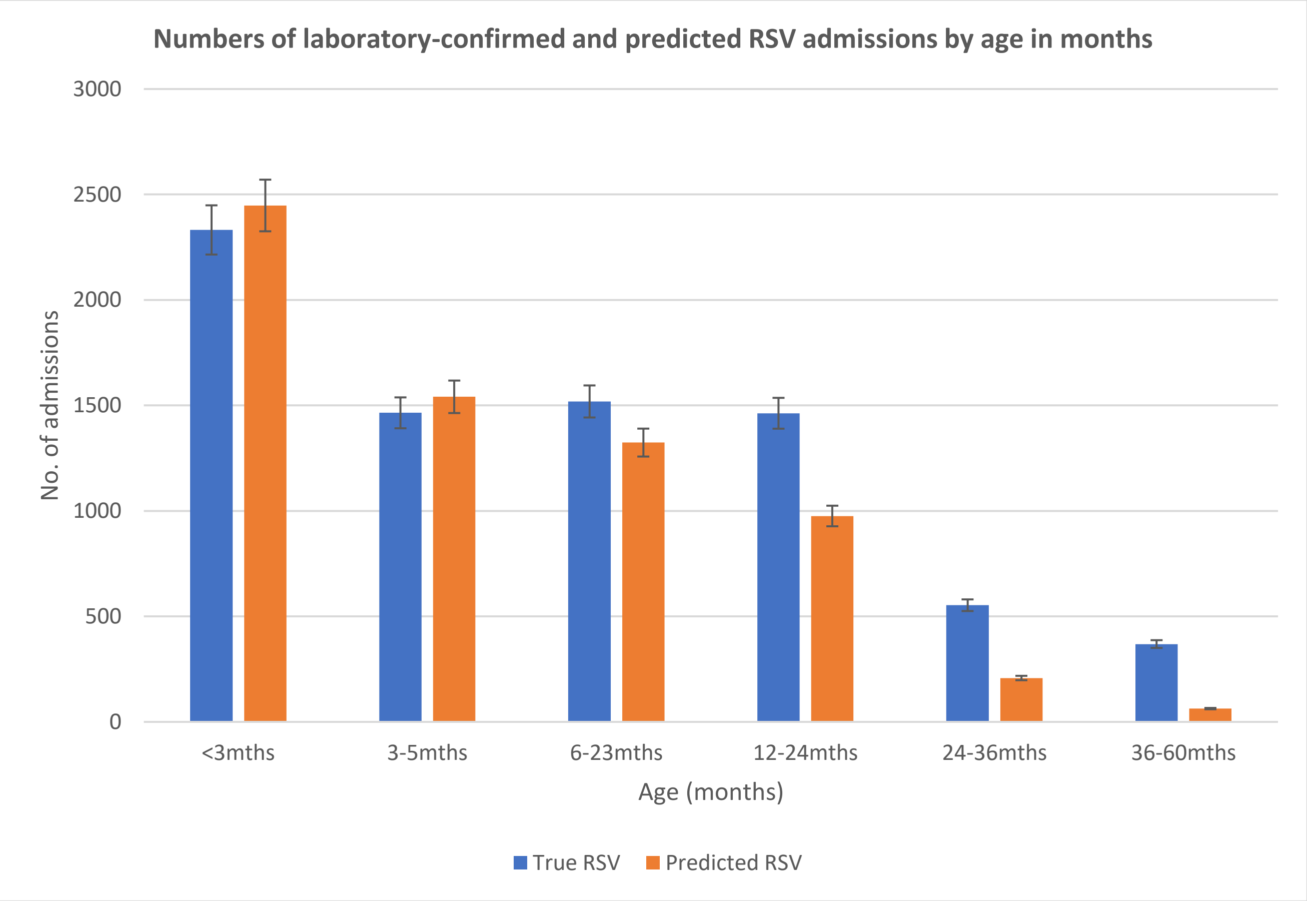

eFigure 4. Number of laboratory-confirmed RSV-positive admissions and the number predicted RSV by the final model, by age at admission

[1] eFigure 4- eFigure 7 presented a detailed look into the model fit of the predictors which indicates whether the model was a good fit for each predictor or under/overestimated the numbers of RSV associated admissions.

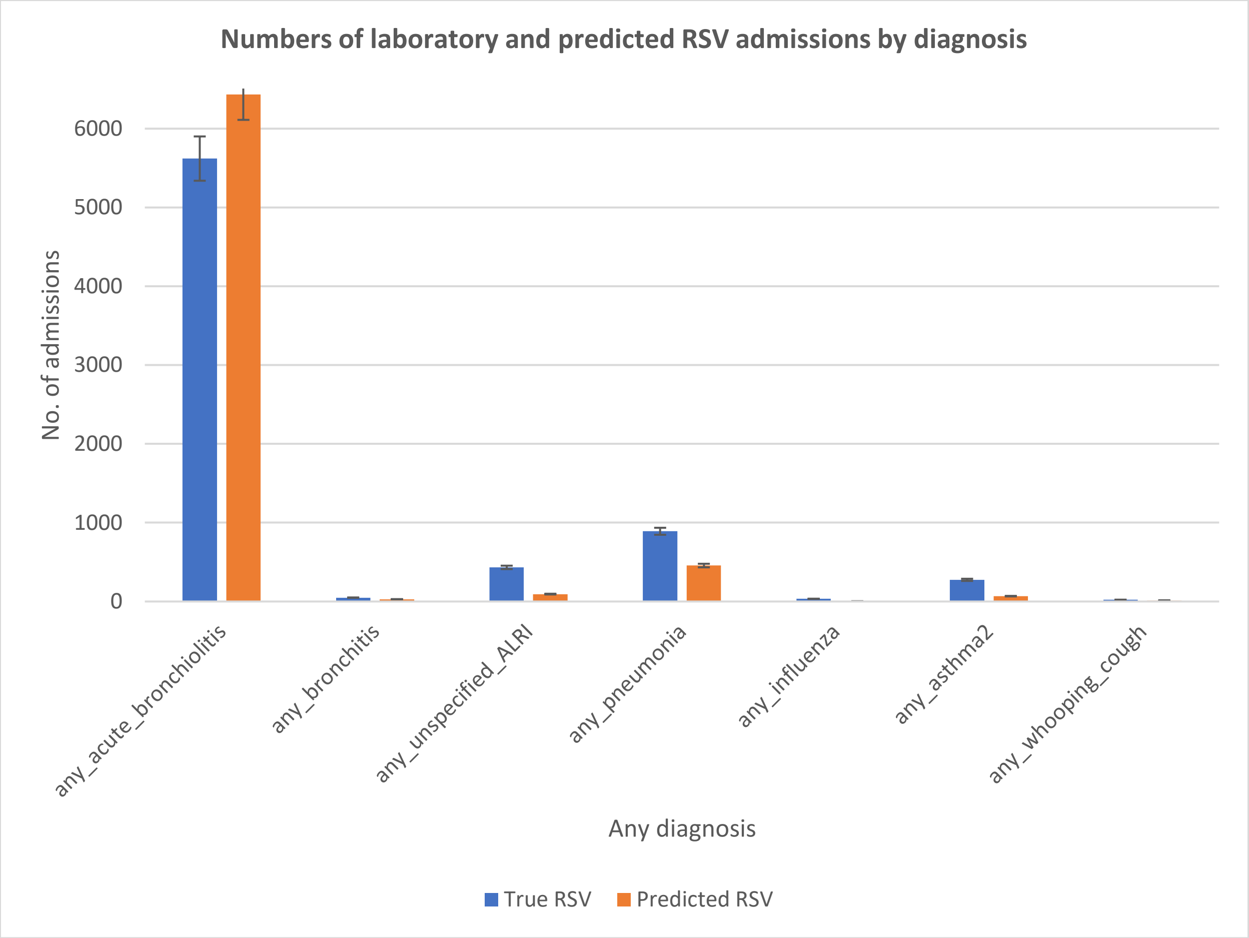

eFigure 5. Number of laboratory-confirmed RSV-positive admissions and the number predicted RSV by the final model, by age diagnosis at admission

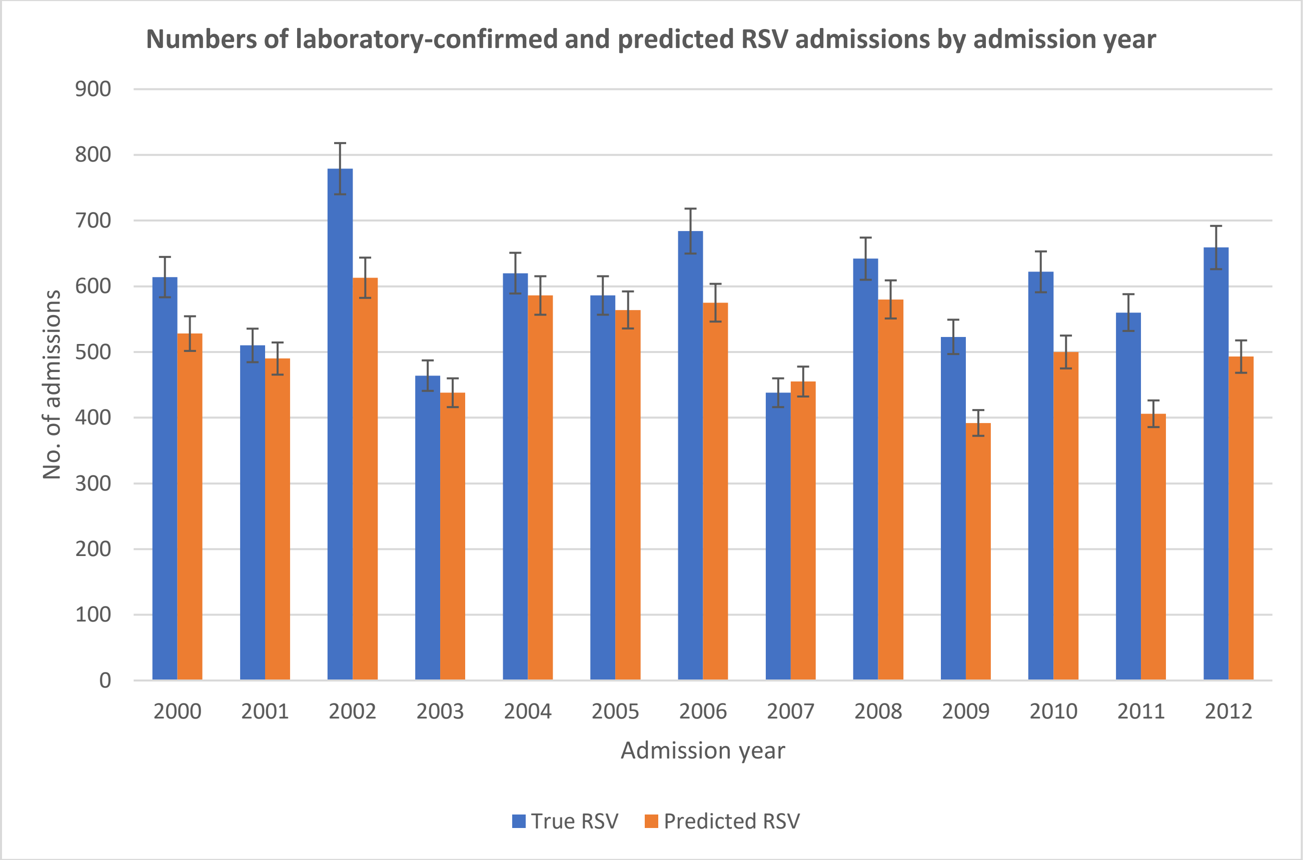

eFigure 6. Number of laboratory-confirmed RSV-positive admissions and the number predicted RSV by the final model, by age admission year

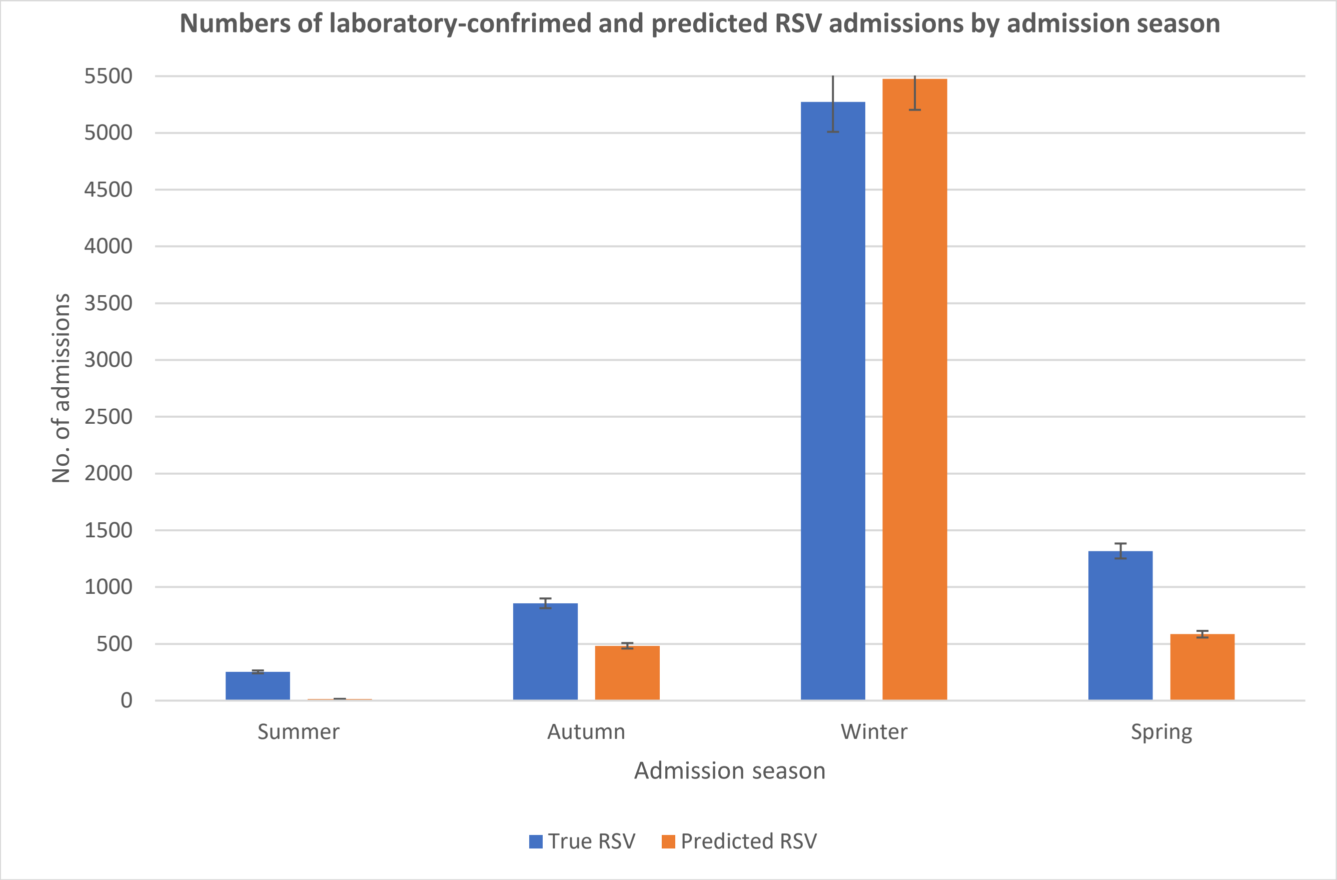

eFigure 7. Number of laboratory-confirmed RSV-positive admissions and the number predicted RSV by the final model, by age admission season

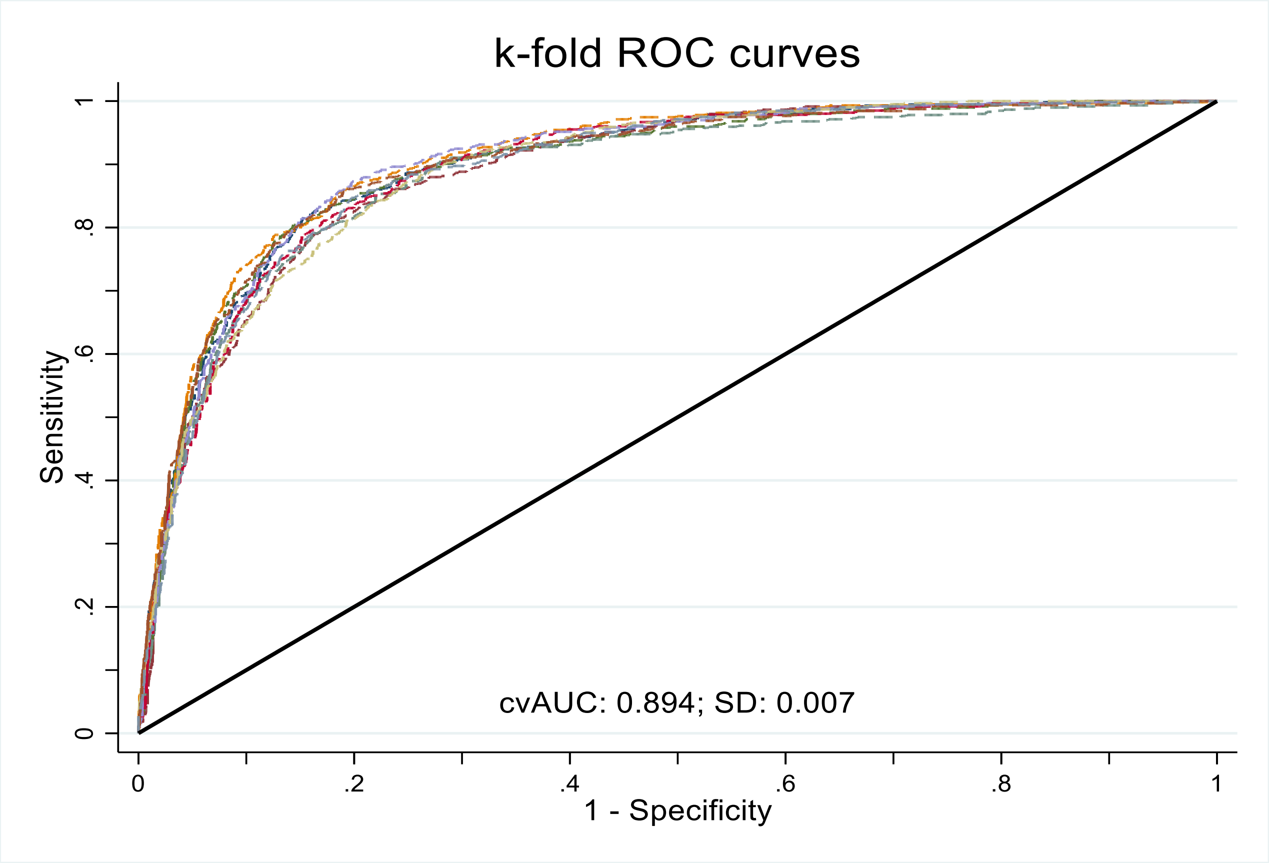

eFigure 8. Receiver-operator curve (ROC) and AUC score computed with 10-fold cross-validation for predicted RSV using immune-fluorescence (IF) test

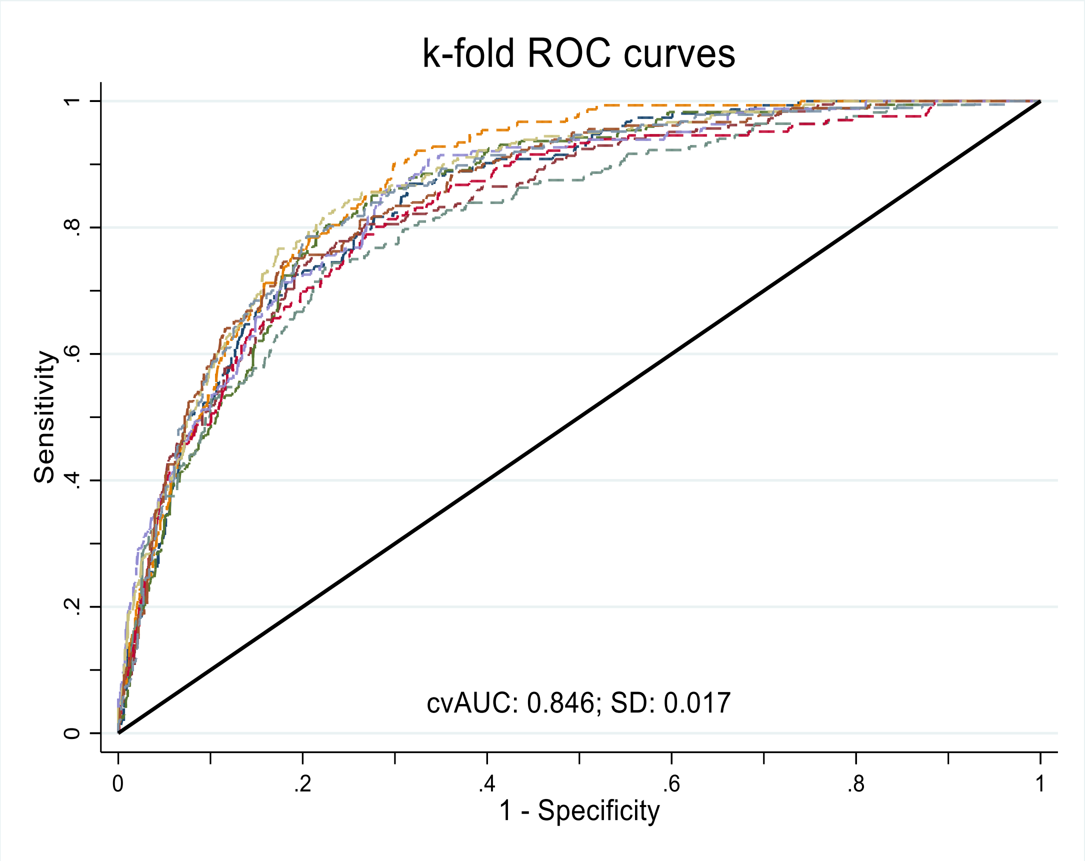

eFigure 9. Receiver-operator curve (ROC) and AUC score computed with 10-fold cross-validation for predicted RSV using PCR test
